# Mild Parkinsonian Signs in the Elderly – Is There an Association with PD? Crossectional Findings in 992 Individuals

**DOI:** 10.1371/journal.pone.0092878

**Published:** 2014-03-27

**Authors:** Stefanie Lerche, Markus Hobert, Kathrin Brockmann, Isabel Wurster, Alexandra Gaenslen, Sandra Hasmann, Gerhard W. Eschweiler, Walter Maetzler, Daniela Berg

**Affiliations:** 1 Center of Neurology, Department of Neurodegeneration and Hertie-Institute for Clinical Brain Research, University of Tübingen, Tübingen, Germany; 2 German Center for Neurodegenerative Diseases, University of Tübingen, Tübingen, Germany; 3 Geriatric Center and Department of Psychiatry and Psychotherapy, University Hospital of Tübingen, Tübingen, Germany; UCL Institute of Neurology, United Kingdom

## Abstract

**Background:**

Mild parkinsonian signs (MPS) are common in the elderly population, and have been associated with vascular diseases, mild cognitive impairment and dementia; however their relation to Parkinson's disease (PD) is unclear. Hypothesizing that individuals with MPS may reflect a pre-stage of PD, i.e. a stage in which the nigrostriatal system is already affected although to a milder degree than at the time of PD diagnosis, aim of this study was to evaluate the similarities between MPS and PD.

**Methods:**

The TREND study is a prospective cross-sectional cohort study in individuals >50 years with biennial assessments designed to identify markers for an earlier diagnosis of Parkinson's and Alzheimer's disease. For this substudy 992 individuals were included for analyses (892 controls, 73 MPS individuals, 27 PD patients). Parameters defining risk of PD (sex, age, positive family history), prodromal markers (hyposmia, REM sleep behavior disorder, depression and autonomic failure) as well as quantitative fine motor, axial motor and cognitive parameters were compared between the three cohorts.

**Results:**

As expected, PD patients differed from controls with regard to 12 of 15 of the assessed parameters. MPS individuals differed significantly from controls in 12 of the PD-associated parameters, but differed from PD only in 5 parameters.

**Conclusion:**

This study shows that individuals with MPS share many prodromal and clinical markers of PD with PD patients, implying that either a common dynamic process or similar constitutional factors occur in MPS individuals and PD patients.

## Introduction

Parkinson's disease (PD) is characterized by the cardinal motor features resting tremor, rigidity and bradykinesia. However, each symptom individually as well as in combination is also highly prevalent in elderly people who do not meet the criteria for Parkinson's disease (PD) [1] or other neurodegenerative diseases [2]–[7]. Therefore they are referred to as mild parkinsonian signs (MPS). MPS are associated with depression [8], hyposmia [9], slight cognitive impairment [10] and dementia [11]. Moreover, in individuals with MPS they are claimed to be a predictor for mortality [12]. These symptoms are also known to occur as “prodromal markers” in Parkinson's disease [13]–[15].

Up to now it is not entirely clear whether individuals with MPS progress to a distinct neurological disease entity and if so, whether they develop primarily PD, Alzheimer's Disease (AD), vascular parkinsonian syndromes or vascular dementia [16], [17]. To solve this issue, there are several studies focusing on the association of MPS with the future development of dementia [3], [5], [7], but little is known about the association with PD. If MPS, at least in a subgroup of individuals, reflect a pre-stage of PD it must be a stage in which the nigrostriatal system is already affected – i.e. an early motor stage. Thus, assuming that the neurodegenerative process in most PD patients indeed affects the central nervous system in an ascending way, as first hypothesized by Braak et al. [18], individuals with MPS should have a similar prodromal phase as PD patients and should also show deficits in Executive Function (EF).

The aim of this study was to evaluate the similarities between MPS and PD. Therefore we investigated the association of MPS with risk factors, prodromal marker and early motor symptoms of PD. To better quantify motor abnormalities we used accelerometer based assessments and an automated device to quantitatively assess fine motor tasks.

## Subjects and Methods

### Study population

For this analysis 1125 participants of the TREND study, including eight individuals who had developed PD at the first follow-up but had no Parkinsonism at baseline and 19 early-stage PD patients from our outpatient clinic were included. The TREND study (**T**übinger evaluation of **R**isk factors for **E**arly detection of **N**eurodegenerative **D**isorders) is a prospective follow-up study in individuals >50 years with biennial assessment until death/autopsy. All participants were pre-screened via telephone interview before study inclusion in 2009/10. Exclusion criteria comprised a reported history of psychiatric diseases (other than unipolar major depression), dementia, epilepsy, stroke, multiple sclerosis, encephalitis and malignancies, intake of antipsychotics and other drugs that may promote Parkinsonian symptoms. For a detailed outline of the TREND study, inclusion and exclusion criteria and baseline assessments see Berg et al.[19]. A large assessment battery with mainly quantitative, unobstrusive measurements designed to be repeated easily and objectively, is being applied. Thus, with regard to neuroimaging only transcranial sonography is applied to the whole cohort. More detailed neuroimaging with more sophisticated techniques are carried out in substudies in parts of the cohort, which, however, is not the focus of this manuscript. All assessments are performed by a group of experienced investigators. To make sure that there is no bias in data acquisition all investigators are blinded to the results of all other examinations.

### Ethics Statement

The study was approved by the ethical committee of the Medical Faculty of the University of Tübingen (Nr. 90/2009BO2), and all subjects gave written informed consent. To rule out compromised capacity/ability to consent we asked participants for known neurodegenerative diseases, including dementia in the pre-screening.

### Final sample

In total we investigated 1144 participants, of whom 27 had a diagnosis of Parkinson's disease, (median age 64 (41–83); 599 males and 548 females). To avoid an influence of additional symptoms on the study outcome we had to exclude several individuals: 51 participants who scored on the Beck Depression Inventory (BDI) >18, as major depression is associated with psychomotor slowness, 45 individuals with traumatic brain injury, 28 individuals who had a stroke within the first two years of the study, 11 non-native speakers, seven with brain tumor, six with Mini-Mental State Examination (MMSE)<25 implying a dementive process, three with negative delta Trail Making Test (TMT) values (see below) and one with a secondary Parkinsonian Syndrome. Thus 992 participants were finally analyzed (median age 64 (41–83); 527 males and 465 females).

### Neurological Examination

Each participant underwent a standardized neurological examination including the motor part of the Unified Parkinsons Disease Rating Scale (UPDRS) by an experienced movement disorder specialist [20]. For the diagnosis of MPS an abbreviated 10-item version of the UPDRS was used [10]. The 10 items included speech, facial expression, tremor at rest (in any body region), rigidity (rated separately in the neck, right arm, left arm, right leg and left leg), posture and body bradykinesia, with each item rated from 0 to 4. A parkinsonian sign score (range = 0 to 40) was calculated for each participant. MPS were defined as present when any one of the following conditions was met: 1) two or more abbreviated UPDRS ratings = 1 or 2) one abbreviated UPDRs rating≥2 or 3) an abbreviated UPDRS rest tremor rating = 1. PD was diagnosed according to the UK Brain Bank clinical diagnostic criteria.[1]

To minimize the variability in the UPDRS rating only raters with several years of experience rated the participants.

### Assessment of risk factors

Each participant underwent a structured medical interview including demographics, medical history and medication.

A family history of PD was recorded according to the criteria of Marder and colleagues [21]. A positive family history was stated to be present if a family member showed at least three parkinsonian signs (tremor at rest, shuffling gait, stooped posture, muscular rigidity) or showed one of these signs and had one of the following: general physician- or neurologist diagnosed PD or positive response to levodopa.

### Assessment of Prodromal markers

#### Impaired olfaction

Olfaction was tested using a 16 Sniffin' sticks battery (BurghartMedizintechnik, Germany) as described by Hummel et al. [22]. According to the suggestion of Hummel and colleagues, less than 75% of correctly identified odours were classified as abnormal (hyposmia).

#### Rapid eye movement sleep behaviour disorder (RBD)

Presence of possible RBD was determined by a self-administered RBD screening questionnaire (RBDSQ).The RBDSQ is a recently developed questionnaire, comprising 10 items to describe the most prominent clinical features of RBD [23].

#### Depression

Depressive symptoms were measured by the Beck Depression Inventory II (BDI) [24]. The BDI is a 21-item self-report questionnaire, ranging from 0 to 60. Based on the BDI-sum, a cut-off score for Major depressive disorder (MDD) was defined as a BDI-score as >18.

#### Autonomic dysfunction

The Unified Multiple System Atrophy Rating Scale (UMSARS) is a validated, disease-specific scale assessing the diverse signs and symptoms in MSA [25]. In the UMSARS part I motor functions of daily living and autonomic functions are evaluated. For this study we built an UMSARS score out of the autonomic questions 9 to 12. In these questions orthostatic symptoms, urinary function, sexual function and bowel function are assessed. Higher scores show more severe autonomic dysfunction.

#### Accelerometer based exams

All subjects performed four single task and two dual task trials. The four single tasks were: 1. walking with habitual speed, 2. walking with maximum speed, 3. checking boxes with maximum speed and 4. subtracting serial 7 s with maximum speed. The two dual task trials were: 1. walking with maximum speed and checking boxes with maximum speed. 2. walking with maximum speed and subtracting serial 7 s with maximum speed. All assessments were performed in an at least 1.5 meters wide corridor allowing obstacle-free 20 meter walks.

Dual task costs were calculated using the following formula according to [26], [27]:
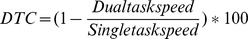
Formula 1: calculation of dual task costs

This formula gives information about the percentage of change compared to the single task value. A positive value indicates a decrease of speed. For detailed information see Hobert et al. [28].

#### Purdue PegBoard

A standard Purdue pegboard (Lafayette Instruments, Lafayette, IN, USA) was used. Participants performed three tasks: right hand, left hand and both hands. Each task consisted of three 30 s trials with 20–30 s inter-trial intervals. During the task, a participant had to pick single pegs from a well, one at a time, using the thumb and index fingers of one hand, and place them in specific holes on the pegboard (e.g. right hand, right column beginning at the top). Subjects were encouraged to place as many pegs as possible and the number of pegs placed on each 30 s trial was recorded. An average of number of pegs placed over the three trials was calculated. Additionally a sum of scores (right hand+ left hand+ both hands) was calculated and used for further analyses.

#### Cognitive assessment

The TMT is a widely used paper-and-pencil task that evaluates the executive functions cognitive flexibility and working memory [29], [30]. The TMT consists of two parts: In TMT Part A subjects have to connect numbers from 1 to 25, which are randomly spread over a sheet of paper, in ascending numerical order. In part B, participants are asked to connect randomly spread numbers (from 1 to 13) and letters (from A to L) in alternating numeric and alphabetical order (1-A-2-B-3-C-…-13-L). In case of an error the examiner draws the attention of the participant to the error, so that the participant completes the task without errors (at the expense of additional time). The maximum time allowed was 300 s. After this time the investigator discontinued the experiment [31]. TMT performance was calculated taking the time needed to perform TMT-B minus the time needed for TMT-A. This delta TMT value prevents possible bias due to differences in upper extremity motor speed, simple sequencing, visual scanning, and psychomotor functioning [29], [30].

### Number of assessed parameters

In sum, we evaluated 15 parameters including risk factors (male gender, positive family history of PD and age>60 years; n = 3); prodromal markers (hyposmia, depression, possible RBD and autonomic dysfunction; n = 4), quantitative motor markers (Purdue PegBoard, four different dual task cost parameters; n = 5) and cognitive parameters (TMT part A, TMT part B and delta TMT B-A; n = 3).

### Statistics

Statistical analysis was performed using JMP10.0.0 (SAS). Due to asymmetric distribution differences between the three groups (Control, MPS and PD) were evaluated either with the Kruskal-Wallis test for non-categorical data, or the chi-square test for categorical data. Differences between two groups were investigated by post-hoc Wilcoxon rank-sum test or by Fishers exact test. Descriptive statistics are given either as median and range for non-categorical data or as percentages for categorical data. Since age, gender, MMSE and BDI were significantly different between the groups and there were correlations between these factors and the performance in motor and cognitive tasks we corrected them by use of a logistic regression model, and assessed significance of each model effect by the likelihood ratio. Differences were considered significant at p<0.05.

## Results

A final total of 992 persons were included in the analysis. MPS were present in 73 (7.4%) participants. None of them fulfilled the criteria of PD. Individuals with MPS were more often male (64% vs. 52%; p<0.05), suffered more often from hyposmia (23% vs. 13%; p<0.05) or possible RBD (19% vs. 9%; p<0.01), had higher depression and autonomic dysfunction scores than controls. With regard to the prodromal markers we could show a correlation between hyposmia and RBD (ρ = 0.159, p<0.0001) (table 1). Details of demographic and clinical variables are supplied in table 2.

**Table 1:** Association between depression, hyposmia and possible RBD.

	RBD	Depression	Hyposmia
RBD	-	26%; ρ = 0.035	32%; ρ = 0.159*
Depression	10%; ρ = 0.035	-	12%; ρ = −0.026
Hyposmia	19%; ρ = 0.159*	19%; ρ = −0.026	-

RBD, Rapid eye movement sleep behavior disorder; ρ, Spearmans Rho.

*p<0.001.

**Table 2:** Demographic and clinical characteristics of participants.

	Controls (n = 892)	MPS (n = 73)	Parkinson (n = 27)	p-value
Sex f/m	429/463	26/47*	10/17	0.07
age	64 (50–83)	67 (54–80)**	66 (41–80)	0.02
Education	13 (9–22)	13 (9–21)	13 (9–19)	0.18
MMSE	29 (25–30)	28 (25–30)	28 (26–30)	0.02
BDI	4 (0–17)	5 (0–17)*	7 (0–17)*	0.02
Hyposmia (%)	104 (13)	15 (23)*	6 (75)**††^a^	0.001
possible RBD (%)	60 (7)	13 (19)**	4 (50)**†^a^	0.001
UMSARS Score	2 (0–10)	2 (0–8)**	4 (4–8)**†† a	0.001
Orthostatic problems (%)	209 (24)	19 (27)	10 (37)	0.23
Urinary dysfunction (%)	331 (37)	38 (52)*	12 (44)	0.04
Sexual dysfunction (%)	447 (58)	49 (73)**	10 (100)**^a^	<0.01
Constipation (%)	124 (14)	13 (18)	7 (26)	0.16
Positive family history PD (%)	114 (13)	10 (14)	3 (11)	0.93
Positive family history AD (%)	226 (26)	11(16)	4 (15)	0.18

Values are presented as median (range) or as quantity (percentage).

BDI, Beck's Depression Inventory; MMSE, Minimental State Examination; MPS, Mild Parkinsonian Signs; RBD, Rapid eye movement sleep behavior disorder.

a:Data not available of all PD patients due to incomplete questionnaire (8 out of 27).

p<0.05: * compared to controls; † compared to MPS.

p<0.01: ** compared to Controls; ††compared to MPS.

**Figure 1:**
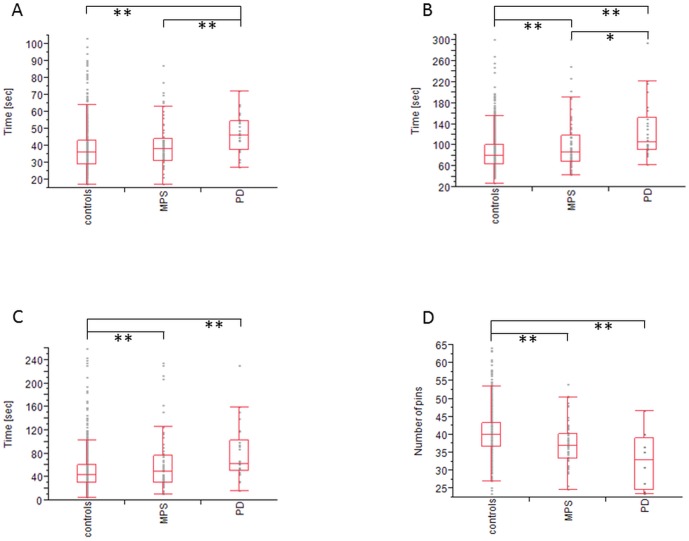
Trail Making test and Purdue PegBoard test results of Controls, individuals with MPS and Parkinson. A) TMT part A. B) TMT part B. C) delta TMT B-A. PD patients were slower in all three TMT tasks compared with controls. Individuals with MPS need more time for completion of TMT part B and in delta TMT B-A than controls but do not differ in the easier TMT part A. In the more complex tasks (B+C) PD patients and individuals with MPS perform similar. D) sumscore of single right hand, single left hand and both hands: In the quantitative fine motor task individuals with MPS as well as PD patients, perform slower than controls. MPS, Mild Parkinsonian Signs; PD, Parkinson's Disease; TMT, Trail Making Test. *: p<0.05; **: p<0.01

Individuals with MPS accomplished the TMT part A (38 s vs. 46 s; p<0.01) and part B (87 s vs. 105 s; p<0.05) faster than PD patients. In contrast they were slower than controls only in the more challenging TMT part B (80 s vs. 87 s; p<0.01) but not in part A. However, assessing delta TMT values, individuals with MPS needed more time than controls (43 s vs. 49 s; p<0.01) and performed similar to PD patients. Finally PD patients had slower results in all three TMT analyses compared to controls (p<0.01). (Figure 1A-C)

Using the PurduePegBoard test, individuals with MPS and PD patients were significantly slower than controls (HC: 40 pins, MPS: 37 pins, PD: 33 pins; each p<0.01). However, individuals with MPS did not differ from PD patients with regard to speed. (Figure 1D)

Under single tasking conditions, habitual walking speed, maximum walking speed, and checking boxes speed individuals with MPS performed worse than controls (each p<0.01). There was no difference between the three groups (controls, MPS and PD) in the subtracting task. (Figure S1A-D)

**Figure 2:**
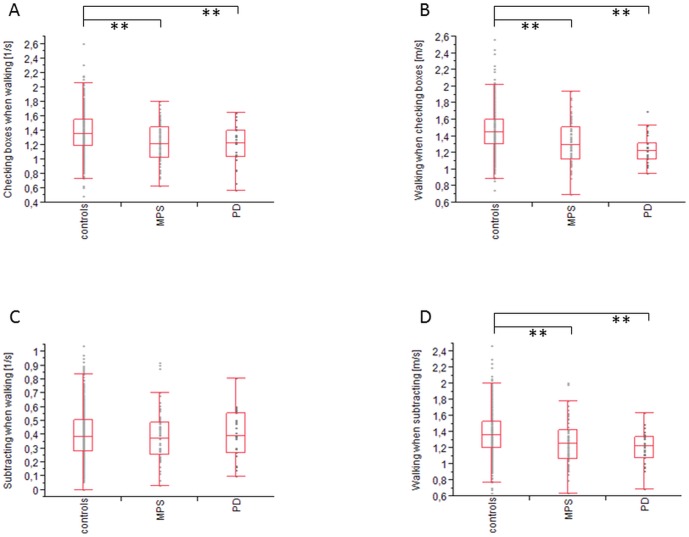
Dual task results of Controls, individuals with MPS and Parkinson's disease. Individuals with MPS and PD patients walk slower and check fewer boxes under dual task conditions compared to controls. There is no speed difference between PD patients and individuals with MPS. MPS, Mild Parkinsonian Signs; PD, Parkinson's Disease *: p<0.05; **: p<0.01

Under dual tasking conditions, checking boxes speed when walking with maximum speed, maximum walking speed when checking boxes and maximum walking speed when subtracting serial 7 s individuals with MPS or PD performed worse than controls p<0.01). There was no difference between individuals with MPS or PD in these tasks. Analog to single tasking there was no difference between the three groups under dual task condition subtracting serial 7 s speed when walking with maximum speed. (Figure 2A–D)

Dual task costs were not significantly different between the investigated groups for checking boxes speed and for subtracting serial 7 s speed. Dual task costs at maximum walking speed when checking boxes and dual task costs at maximum walking speed when subtracting serial 7 s were higher in individuals with MPS and PD patients. Detailed data are shown in table 3.

**Table 3:** Dual task costs.

	Controls (n = 892)	MPS (n = 73)	Parkinson (n = 27)	p-value
Walking when checking boxes [%]	10.9 (−101.9–57.1)	14.3 (−44.8–48.4)**	23.9 (−16.0–49.0)**††	<0.001
Checking boxes when walking [%]	12.8 (−94.2–70.6)	17.1 (32.1–52.4)	17.6 (−41.9–65.2)	0.11
Walking when subtracting [%]	16.2 (−72.6–66.5)	18.7 (−49.6–57.5)^a^	24.3 (−1.3–55.2)*	0.02
Subtracting when walking [%]	−1.8 (−368.5–100)	−0.7 (−175.7–91.3)	9.1 (−339.7–70.4)	0.89

MPS, Mild Parkinsonian Signs.

p<0.10: ^a^compared to controls.

p<0.05: *compared to controls.

p<0.01: **compared to Controls; †† compared to MPS.

In total PD patients differed from controls with regard to 12 of the 15 parameters. MPS individuals also differed significantly from controls in 12 of the PD-associated parameters, but differed from PD only in five parameters.

In contrast to individuals with MPS or PD no difference could be detected in individuals with RBD, depression or hyposmia with regard to the UMSARS scores or the time needed for delta TMT compared to controls without any of these prodromal markers.

## Discussion

In this study we investigated whether individuals with MPS show a similar pattern of prodromal markers as individuals with PD.

**Figure 3:**
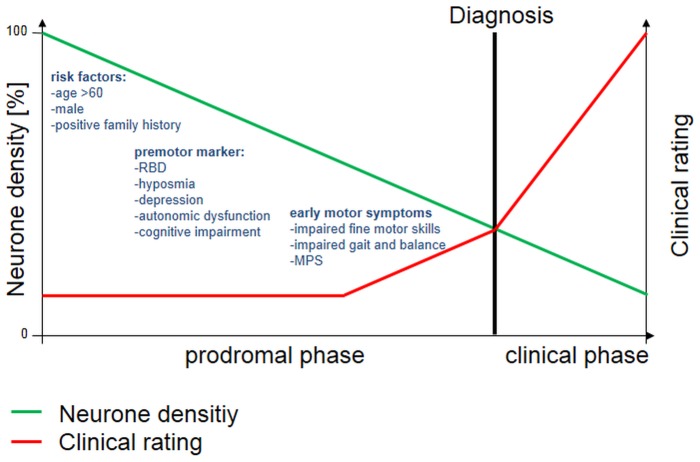
Evaluated risk factors, premotor markers and early motor symptoms in a model, based on the hypothesis of Braak et al. [18]. MPS, Mild Parkinsonian Signs

According to anatomical patterns of Lewy deposition in the Braak staging system [18] we hypothezised that the prodromal phase of PD is characterized by the occurrence of non-motor and/or slight motor features which occur well before onset of typical motor symptoms [15], [32]. In the case of occurrence of both non-motor and early motor symptoms, early motor symptoms emerge often later than the non-motor symptoms [15], [33], [34] – suggesting that the alterations in the nigrostriatal system follow alterations of other (in general lower) parts of the nervous system (Figure 3). We found that individuals with MPS, like PD patients, have a greater frequency of depression, hyposmia, possible RBD and autonomic dysfunction than controls. The association of MPS with depression and hyposmia has been shown in previous studies [8], [9]._ENREF_10_ENREF_35 The association with possible RBD reported here is new to our knowledge and supports the association of MPS with PD, as RBD is known to be the factor with the highest relative risk of any currently known clinical marker for the future development of the clinical phenotype of a synucleinopathy [33].

In addition to the non-motor prodromal features of PD we found in individuals with MPS specific patterns of motor impairment similar to the patterns observed in PD. With regard to bradykinesia of fine motor skills, which is not included in the diagnostic criteria for MPS although it is a central element of PD diagnosis, both groups were equally slow and slower than controls. In addition, we observed that individuals with MPS also walk slower in the single walking tasks compared to controls. These movement impairments may indicate an already affected nigrostriatal system in individuals with MPS.

Impairment of executive function is an important marker for prodromal PD, as it may be challenged under specific conditions unrevealing a fragile system. Initially we used a more simple cognition test: the TMT part A, which is a test for visual search and motor speed skills. In this simple test and also in single task subtracting serial 7s, individuals with MPS did not differ from controls. More challenging tests like the TMT part B - which is a test for complex visual scanning and cognitive flexibility – and the delta TMT B-A revealed significant differences of individuals with MPS and PD patients compared to controls. The same was found for three of the four dual-task conditions. Especially in the latter three conditions individuals with MPS performed at a level similar to PD patients. In addition individuals with MPS and PD patients showed higher dual task costs, which were driven by a deterioration of the walking tasks but they performed as well as controls in checking boxes and subtracting serial 7s tasks. This may be explained by the following consideration: walking is not a simple automatic function that is governed solely by subcortical structures, it may constitute a much more complex task requiring conscious attention and ongoing cognitive processing [35]. Therefore, slowdown in gait speed during dual task conditions is natural because it reduces the risk of falls. It has been shown that healthy individuals follow a posture first strategy [36], [37]._ENREF_37_ENREF_37 Individuals with MPS and PD patients suffer from cognitive inflexibility and therefore may have to slow down their gait speed to a larger extend to achieve the desired cognitive performance and avoid falls.

Taken together, our data from various tests suggest that individuals with MPS, like PD patients, differ strikingly from controls but share a substantial number of features with PD patients. They share many risk factors and prodromal markers of PD, suggesting that either a common dynamic process or similar constitutional factors occur in MPS individuals and PD patients. Because there were no associations in individuals with RBD, hyposmia or depression with autonomic dysfunction or impaired EF the effects detected in MPS seem not to be driven by additional prodromal symptoms.

This is, to the best of our knowledge, the first study demonstrating an association of MPS with PD not only in motor features but also in risk and prodromal markers. However, not all individuals with MPS will develop PD. Some may continue to present MPS all their life, others may develop AD, other forms of Lewy body diseases or a vascular neuropathology. Despite the uncertain future of individuals with MPS, our study indicates that they may form an important group for studying the prodromal phase of neurodegenerative diseases. To better estimate which combination of markers in individuals with MPS may indicate best those who are in a process of neurodegeneration, finally leading to clinical PD, AD or other neurodegenerative diseases, it is essential to investigate individuals with MPS in prospective longitudinal studies.

### Limitations

A major limitation of this study is that the actual definition of MPS does not include a separate rating of bradykinesia of finger/hand movements which is an essential part in PD diagnosis. To address this issue without changing the MPS definition by including additional UPDRS items we evaluated finger/hand movements with the Purdue Pegboard test and showed that MPS is not only associated with whole body bradykinesia but also with distinct bradykinesia finger/hand movements in an objective task.

Another limitation of this study is the diagnosis of RBD which was made according to a questionnaire and not by polysomnography. Although the used questionnaire has high sensitivity and specificity values [23] it is possible that there are some false positives. We therefore used the term “possible RBD”.

Furthermore this study is a crossectional analysis. Therefore, these suggestive results have to be proven longitudinal follow ups of this and other cohorts.

## Supporting Information

Figure S1 **Single task results of Controls, individuals with MPS and parkinson.** Individuals with MPS walk slower, check fewer boxes per second and make fewer subtractions per second than controls. MPS, Mild Parkinsonian Signs; PD, Parkinson's Disease. *: p<0.05; **: p<0.01.
(TIF)
