# Supplementary figures and images for: Mild Parkinsonian Signs in the Elderly – Is There an Association with PD? Crossectional Findings in 992 Individuals
Source: PLoS One. 2014 Mar 27;9(3):e92878. doi: 10.1371/journal.pone.0092878 (PMC3968033; doi:10.1371/journal.pone.0092878)

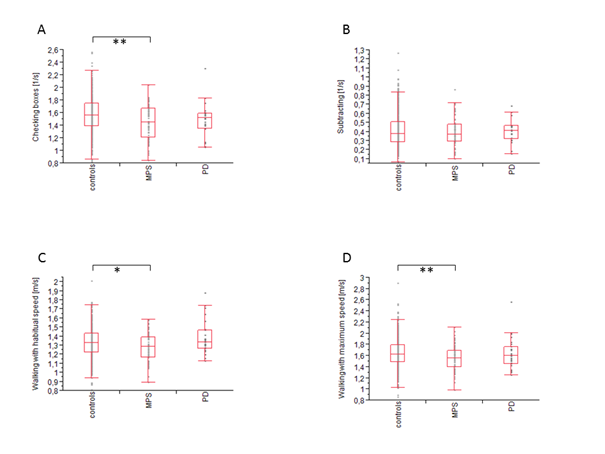

Supplement: Figure S1 — Single task results of Controls, individuals with MPS and parkinson. Individuals with MPS walk slower, check fewer boxes per second and make fewer subtractions per second than controls. MPS, Mild Parkinsonian Signs; PD, Parkinson's Disease. *: p<0.05; **: p<0.01. (TIF) [file pone.0092878.s001.tif]
